# Normal values of femoral head coverage angles on three-dimensional computed tomography images

**DOI:** 10.1371/journal.pone.0347008

**Published:** 2026-10-07

**Authors:** Seohyun Choi, Seon Min Lee, Young Jae Kim, Yong-Cheol Yoon, Kwang Gi Kim

**Affiliations:** 1 Department of Medicine, College of Medicine, Gachon University, Incheon, Republic of Korea; 2 Medical Devices R&D Center, Gachon University Gil Medical Center, Incheon, Republic of Korea; 3 Department of Biohealth & Medical Engineering, Gachon University, Seongnam-si, Gyeonggi-do, Republic of Korea; 4 Biomedical & Convergence Institute, Gil Medical Center, Gachon University, Incheon, Republic of Korea; 5 Orthopedic Trauma Division, Trauma Center, Gachon University College of Medicine, Namdong-gu, Incheon, Republic of Korea; 6 Department of Biomedical Engineering, Gil Medical Center, College of Medicine, Gachon University, Incheon, Republic of Korea; Gdańsk University of Technology: Politechnika Gdanska, POLAND

## Abstract

**Introduction:**

Identifying novel diagnostic parameters is essential to address the inaccuracies related to femoral head coverage angle measurements using conventional methods. This study aimed to propose new femoral head coverage landmarks on 3D pelvic CT images in a Korean population and to present reference values based on these landmarks.

**Methods:**

This single-center retrospective cohort study defined the center, lateral, anterior, and posterior margins of the acetabulum from three-dimensional (3D) pelvic computed tomography (CT) scans of healthy Korean individuals. The lateral center-edge angle (LCEA), anterior center-edge angle (ACEA), posterior center-edge angle (PCEA), anterior acetabular sector angle (AASA), and posterior acetabular sector angle (PASA) were measured in 3D space.

**Results:**

The normal values for 3D LCEA in Korean males and females were 34.8° (SD = 8.3°) and 32.0° (SD = 6.9°), respectively. The 3D angles measured showed significant variations according to sex and age, with center-edge angles (LCEA, ACEA, and PCEA) generally increasing with age, whereas sector angles (AASA and PASA) showed a decreasing trend.

**Conclusion:**

The standardized values and statistical findings based on sex and age provided in this study offer valuable references for guiding surgical interventions in the acetabulum. Also, the proposed method yielded more likely to be reliable results than traditional two-dimensional methods with substantial differences.

## Introduction

The acetabular femoral head coverage angle is a critical factor for maintaining hip joint stability and long-term functionality and plays a key role in diagnosing various hip conditions and strategizing treatments, such as total hip arthroplasty. The angle serves as a key diagnostic parameter for structural hip diseases, including developmental dysplasia of the hip. Accurate diagnosis and timely intervention are essential for preserving hip joint function, preventing complications such as dislocation, and distributing loads evenly to minimize joint wear and damage. Moreover, proper coverage ensures sufficient range of motion, prevents issues such as femoroacetabular impingement, and can mitigate the progression of arthritis [1]. The prevalence of acetabular fractures has been increasing globally. Lommerse et al. reported that the total incidence in the Dutch population increased by 99% in the span of ten years [2] and Park et al. reported a 28% rise in Korean patients in the span of eleven years [3]. Given that they are often caused by high-impact trauma, the need for quick and precise surgical intervention is considerably high [4], which requires comprehensive understanding of the structure of the acetabulum.

Previous studies have used conventional methods for measuring femoral head coverage angles using two-dimensional medical imaging modalities, such as radiography or two-dimensional (2D) computed tomography (CT). Several studies have established normal values or conducted statistical analyses of angles such as the Wiberg angle, which is measured based on the bony lateral edge of the acetabulum. These studies have reported variations in angles, including the lateral center-edge angle (LCEA), across different sexes and age groups. For instance, Miyasaki et al. analyzed femoral head coverage among 120 individuals and reported that coverage tended to increase with age [5]. Hawellek et al. measured six parameters representing the shape of the acetabulum and found significant sex and age differences in LCEA [6]. Studies on single ethnic groups or comparisons between ethnic groups have reported normal LCEA values. For instance, Shi et al. reported a standard LCEA of 32.8° in Chinese adults [7], and Umer et al. reported a standard LCEA of 31.2° in Singaporeans [8]. Tuğrul et al. measured LCEA values in children and adolescents aged 5–14, suggesting that LCEA values exceeding 19°–23° are considered normal, depending on sex and age [9]. Edwards et al. analyzed the differences in hip morphology among four ethnic groups and reported smaller femoral head coverage in Chinese individuals and significant sex differences in LCEA [10].

However, the angles measured using 2D images are prone to errors because they measure the angle projected on a plane. Ogata et al. reported that the use of the Wiberg angle to analyze hip dysplasia in children may be inaccurate [11]. Furthermore, femoral head coverage measured on a 2D image is sensitive to factors such as the pelvic sagittal inclination (PSI), which can vary depending on the patient’s position during imaging, compared to 3D imaging [1,12,13]. In addition, 2D images may not adequately visualize the key anatomical structures such as acetabular walls [14,15], while examination in 3D images before operation showed high accuracy in defining acetabular morphology [16]. Therefore, it is necessary to develop and standardize more reliable indicators using 3D imaging.

Studies have visualized and quantified the femoral head coverage in three-dimensional (3D) images by segmenting the areas of the femoral head and acetabulum. Uemura et al. estimated femoral head coverage using a 3D surface model [13], and Martin et al. proposed a method for finding acetabular abnormalities by assessing the geometric structure of the femoral head [17]. Upasani et al. manually selected the acetabular area and assessed acetabular morphology on 3D images [18]. However, generating coverage areas, whether automatically or manually, requires labor-intensive and time-consuming processes, such as segmenting the lunate surface and femoral head. Additionally, errors may occur when considering the labrum and cartilage, and this method is sensitive to the resolution of CT images, making it less feasible for situations requiring immediate surgical interventions. Furthermore, owing to the limited sample sizes in these studies, it is difficult to adopt their findings as standard reference values.

Therefore, it is necessary to develop a quicker and simpler method than those of the 3D segmenting-based methods, which provides more reliable angle measurements compared to those obtained using 2D images. Herein, we proposed a method for measuring the femoral head coverage angle using 3D CT images and aimed to report reference values for the Korean population. For the reference values measured using the proposed method, we also sought to present the results of statistical analyses according to age and sex. This method is less susceptible to imaging angle variations and is easier to replicate. To the best of our knowledge, techniques or standard values for measuring femoral head coverage angles on 3D images have not yet been established. In this study, we analyzed the 3D pelvic scans of healthy Korean individuals and aimed to present values that differed from the conventional 2D measurements. By providing more reliable diagnostic criteria, these findings will contribute to more accurate diagnosis of structural hip diseases and improve treatment and prevention of acetabular fractures.

## Methods

### CT imaging data

Data from individuals who had undergone 3D pelvic CT scans at the Gachon University Gil Medical Center between December 1, 2015, and January 31, 2024, were collected. The data have been accessed for research purposes since September 1, 2024, and were fully anonymized to ensure that individuals cannot be identified. Both men and women of single ethnicity were included in this study. Individuals aged <20 years or >70 years and those with a history of pelvic or acetabular trauma, or congenital or acquired deformities were excluded.

This study obtained approval from the Institutional Review Board of the Gil Medical Center (approval no. GBIRB2024−251). All procedures were performed in accordance with the ethical standards of the institution, and the 1964 Declaration of Helsinki and its later amendments. Because of the retrospective nature of the study, the need to obtain informed consent was waived for the individual participants by the Institutional Review Board.

The pelvic 3D CT data comprised images from the axial, sagittal, and coronal planes. All CT data were acquired using Siemens Healthineers syngo.via VB60A CT scanner (Siemens Healthineers AG, Germany), with a tube peak voltage of 120 kVp and B60s reconstruction kernel. The voxel size was set to 1.55 mm^3^ (x-axis 0.72 mm, y-axis 0.72 mm, z-axis 3.00 mm), and Hounsfield Units (HU) were used to measure CT grayscale values. All CT data adhered to the DICOM (Digital Imaging and Communications in Medicine) protocol for standardization and compatibility.

All 3D CT images were analyzed using a picture archiving and communications system (PACS) and AVIEW software (version 1.1.44.17-win; Coreline Soft, Korea).

The LCEA, anterior center-edge angle (ACEA), posterior center-edge angle (PCEA), anterior acetabular sector angle (AASA), and posterior acetabular sector angle (PASA) were selected as parameters of femoral head coverage (Fig 1). The LCEA is the angle between the vertical axis of the pelvis and the line connecting the center of the femoral head to the lateral margin of the acetabulum [19 20]. ACEA is the angle between the vertical axis of the pelvis and the line connecting the center of the femoral head to the anterior margin of the acetabulum [19,21]. PCEA is the angle between the vertical axis of the pelvis and the line connecting the center of the femoral head to the posterior margin of the acetabulum [21]. The AASA is the angle between the horizontal axis of the pelvis and the line connecting the center of the femoral head to the anterior margin of the acetabulum [5]. PASA is the angle between the horizontal axis of the pelvis and the line connecting the center of the femoral head to the posterior margin of the acetabulum [5].

**Fig 1:**
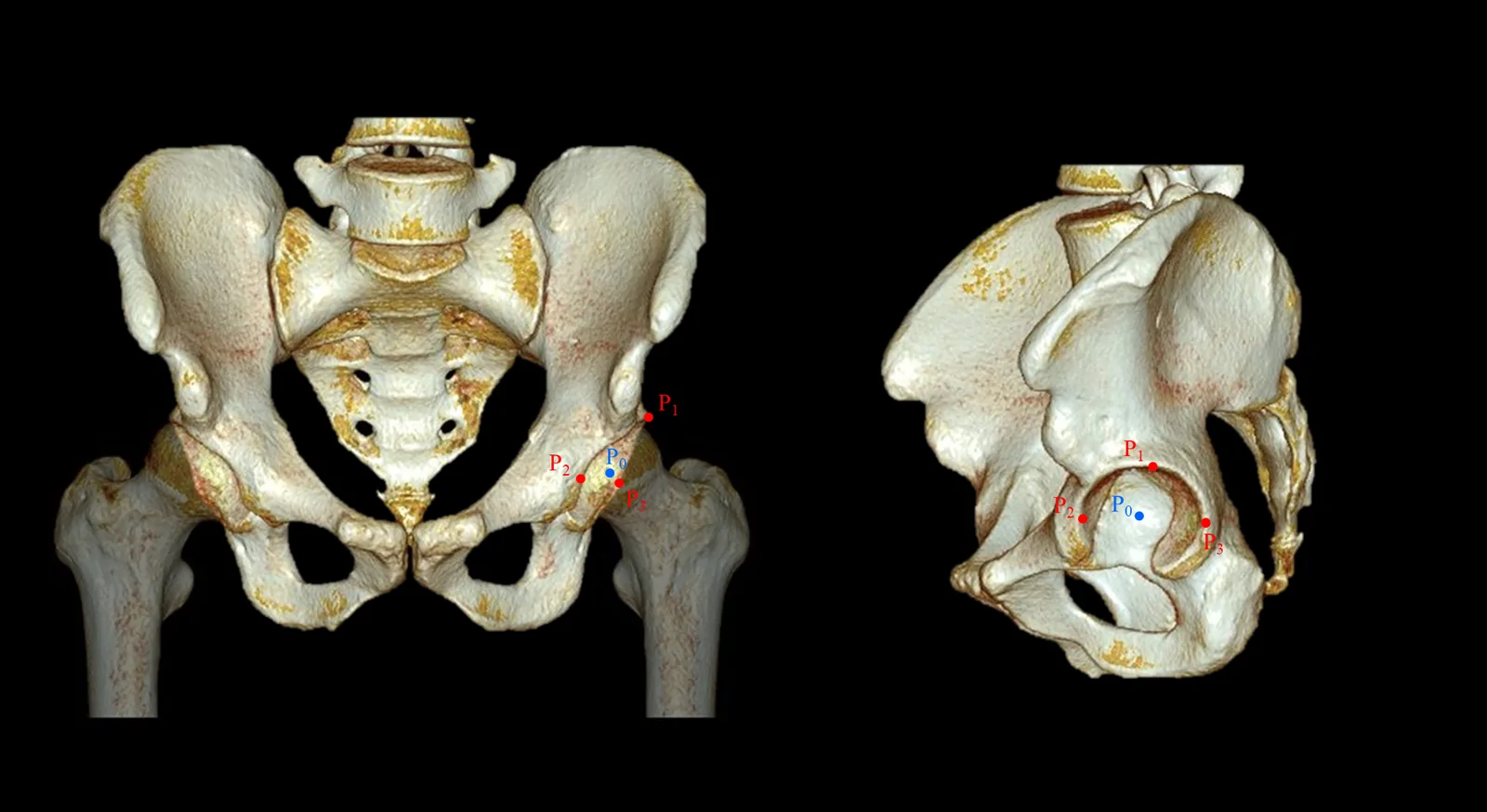
Illustration of angle measurement on 3D images of a pelvis. Positions of P_0_, P_1_, P_2_, and P_3_ and LCEA, ACEA, PCEA, AASA, and PASA are depicted on 3D images of a pelvis.

To measure these angles, four anatomical landmarks were identified: center of acetabulum (P_0_), lateral acetabular margin (P_1_), anterior acetabular margin (P_2_), and posterior acetabular margin (P_3_). Figs 2 and 3 illustrate the positions of these landmarks. All anatomical landmarks were manually placed by medical professionals based on the axial, sagittal, and coronal planes. All anatomical landmark annotations were cross-checked by two board-certified orthopedic surgeons to reduce inter-annotator variability. The axial, sagittal, and coronal planes on which each landmark was located were confirmed, and the coordinates representing each plane were obtained using the AVIEW software, which provided the x, y, and z coordinates on the 3D CT scan.

**Fig 2:**
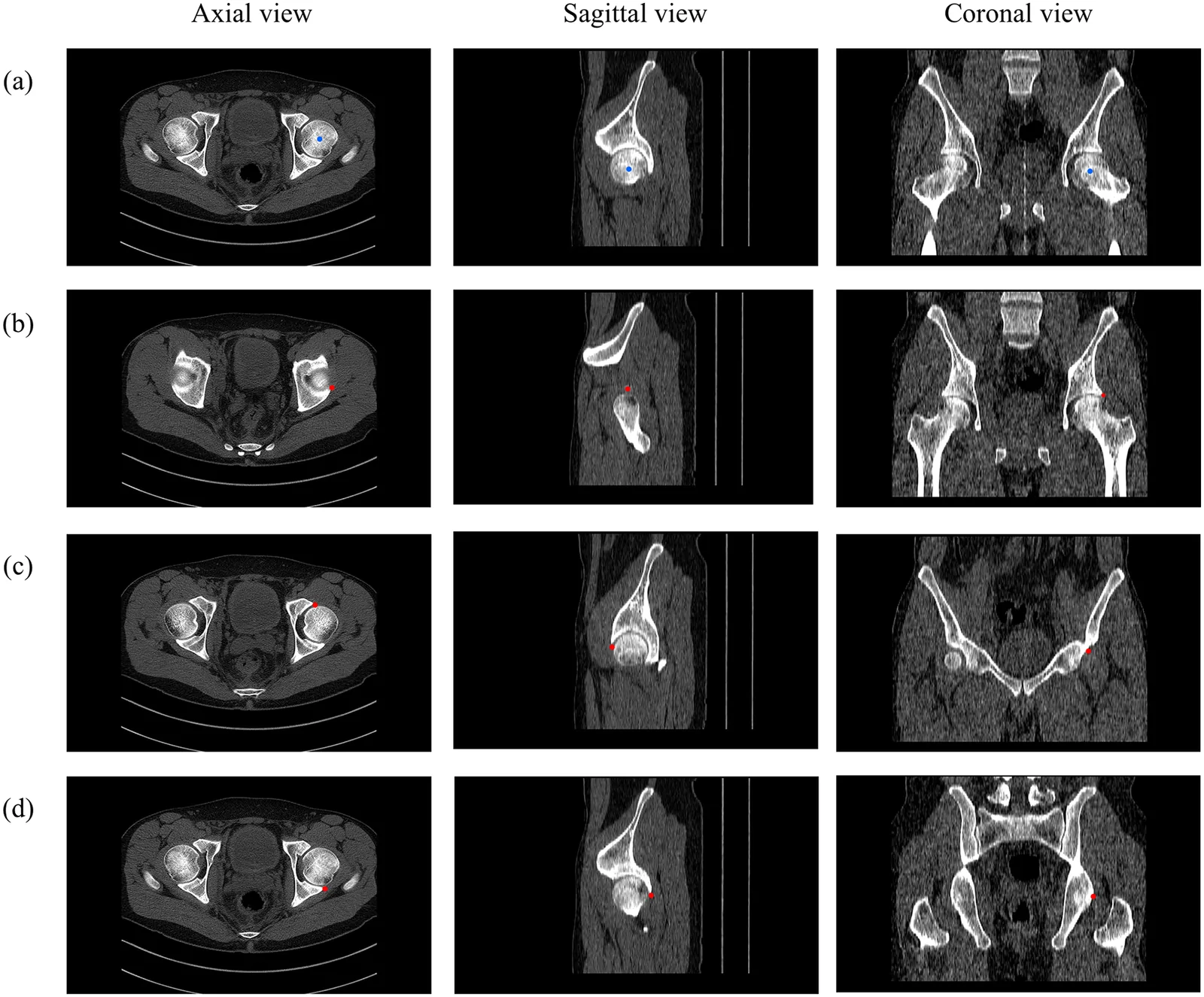
Landmarks for analyzing femoral head coverage. Four landmarks (P_0_: center of acetabulum, P_1_: lateral acetabular margin, P_2_: anterior acetabular margin, P_3_: posterior acetabular margin) were plotted and labeled on three-dimensional computed topography of the pelvis and the bilateral femur in an anterior view (left) and an 18° angle from the complete lateral view (right). On the left panel, image transparencies of the bilateral femur have been adjusted to enhance the visibility of the acetabulum.

**Fig 3:**
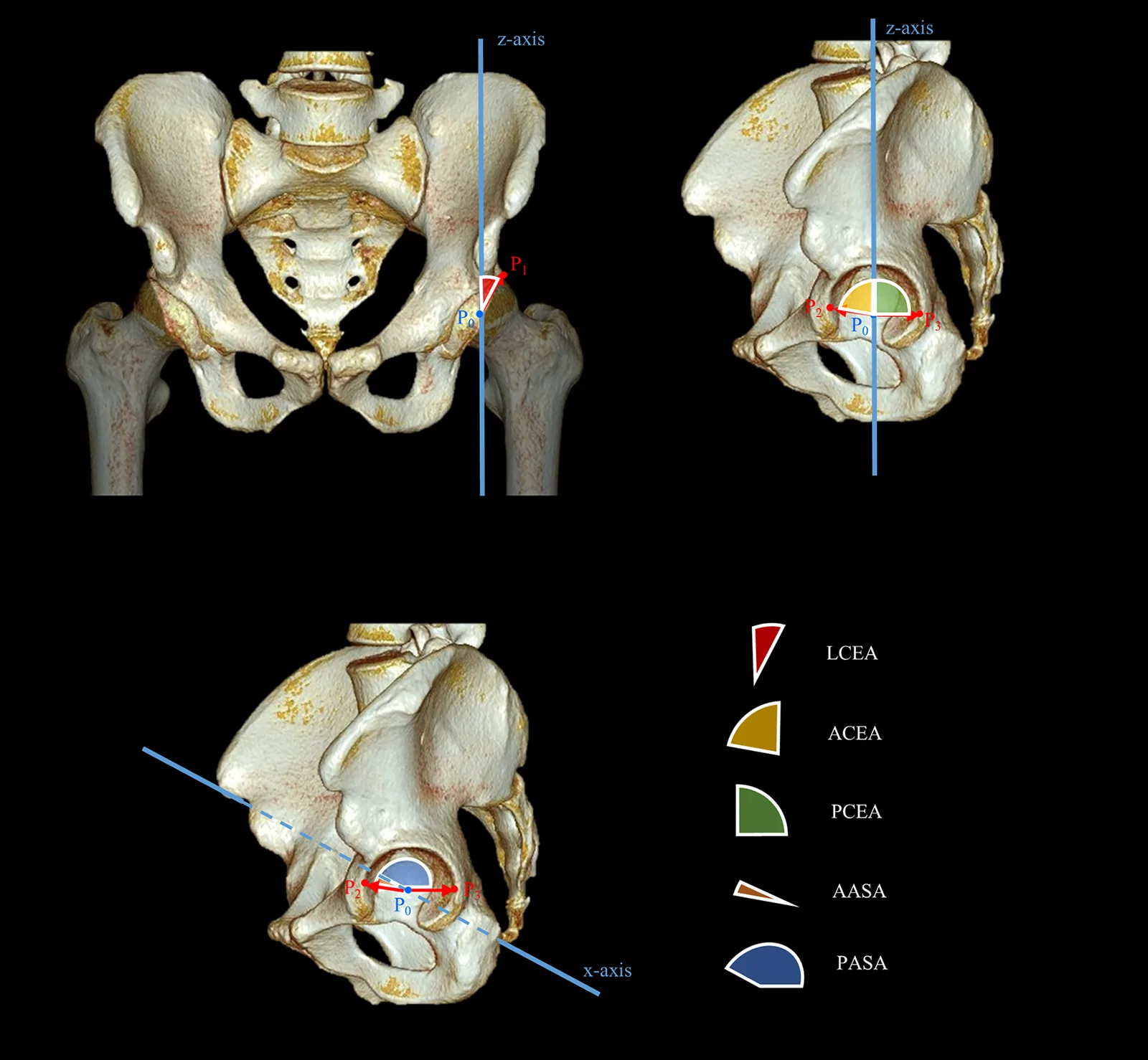
Landmarks for analyzing femoral head coverage on axial, sagittal, and coronal views of the acetabulum. (a) P_0_: center of acetabulum, (b) P_1_: lateral acetabular margin, (c) P_2_: anterior acetabular margin, (d) P_3_: posterior acetabular margin.

P_0_ was defined as the center of the spherical femoral head, determined by selecting one plane each of the coronal, sagittal, and axial views that contained the largest slice of the sphere and pinpointing the center of those slices.
P_1_ was determined as the most lateral point of the acetabular rim, which was identified by starting with the most lateral sagittal plane and progressively scanning more medial planes until the cross-section of the ilium that touches the acetabular head appeared. This point was marked as P_1_.
P_2_ was defined as the most anterior point of the acetabular rim. This was done by starting with the most anterior coronal plane and progressively scanning more posterior planes, selecting the plane immediately before the first one where the cross-section of the femoral head appeared. The coronal view was then fixed, and the axial and sagittal views were adjusted to ensure that P_2_ was accurately positioned on the acetabular rim where it met the femoral head.
P_3_ was defined as the most posterior point of the acetabular rim, identified by starting with the most posterior coronal plane and progressively scanning more anterior planes, selecting the plane immediately before the first one where the cross-section of the femoral head appeared. The coronal view was then fixed, and the axial and sagittal views were adjusted to ensure that P_3_ was positioned on the acetabular rim, where it met the femoral head.

To visualize the parameters, the positions of P_0_, P_1_, P_2_, and P_3_ were displayed on a 3D rendering of the pelvis. Additionally, the vectors used to derive the angles, along with the corresponding angles, were visualized on the image.

### Analysis of femoral head coverage on 3D-CT images

This study analyzed femoral head coverage using 3D coordinates derived from anatomical landmarks. The landmarks P_0_ to P_3_ were represented as 3D coordinates in the form of (x, y, z), which were used to measure the femoral head coverage angles. The angles were calculated by determining the cosine values of the angles between the vector connecting P_0_ to each of the other points and either the x-axis (for the AASA and PASA) or the z-axis (for the LCEA, ACEA, and PCEA). The cosine values were then converted to radians using the ‘arccos*’* function from the Numpy library, and subsequently to degrees using the ‘degrees’ function. The derived angles were evaluated to determine whether they correctly reflected the geometric relationship between P_0_ and the respective margins. The method for calculating each angle based on landmark coordinates is summarized in Figs 1 and 4.

**Fig 4:**
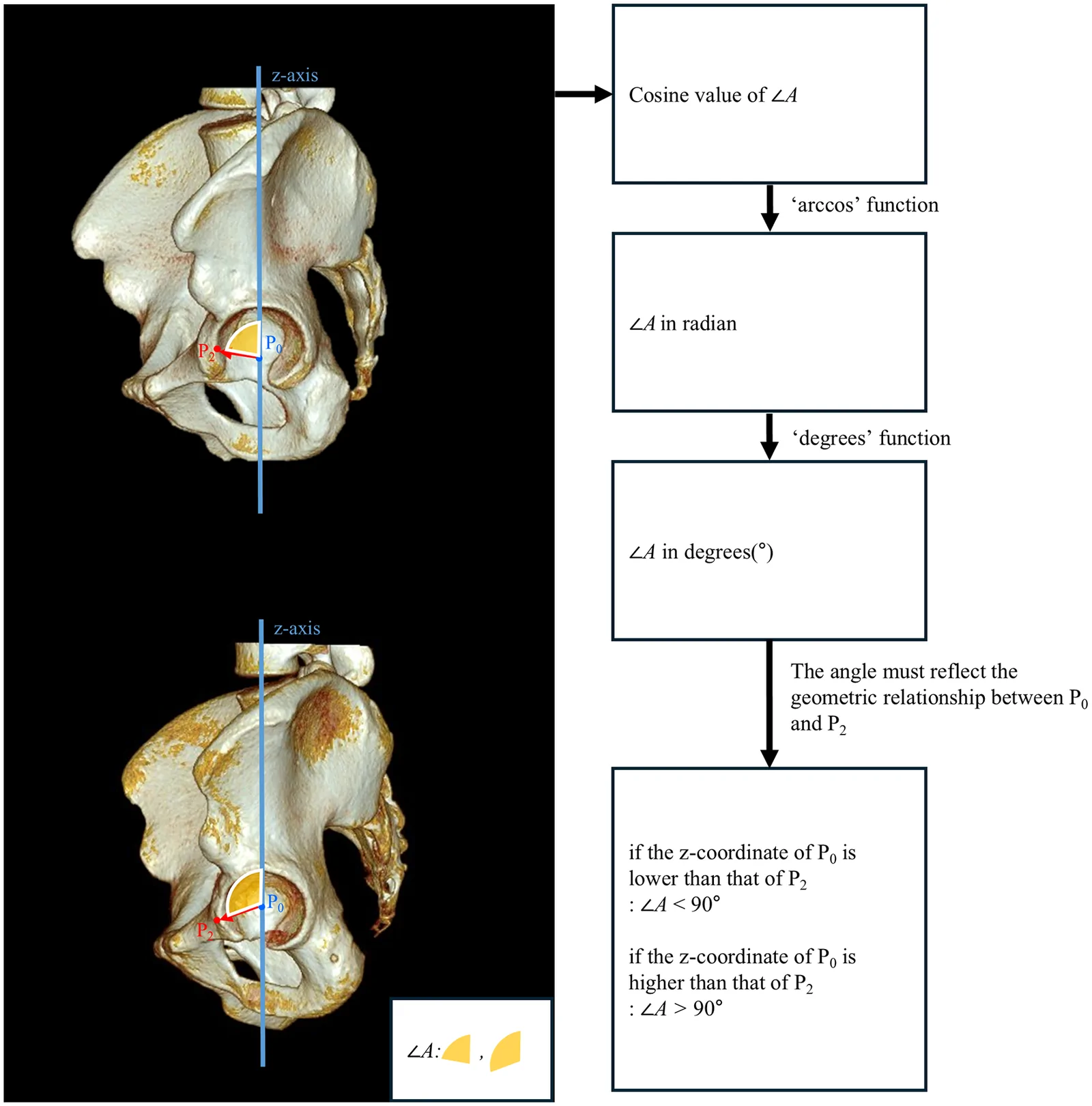
Calculation of the ACEA angle. The angle was calculated by converting the cosine value of the targeted angle into radians and then into degrees (°). ACEA angles were evaluated to determine whether they correctly reflected the geometric relationship of P_0_ and P_2_. Other parameters were calculated using the same method.

### Statistical analysis

Patients were stratified into male and female groups and then classified by age (<50 and ≥50) in line with clinical findings regarding clinical evidence [22,23] and relevant studies [5,24].

All data processing and statistical analyses were performed using functions from the SciPy library (version 1.10.1), Pandas library (version 2.0.3), and the Statsmodels library (version 0.14.4) in Python 3.8.0. Continuous variables are presented as means, standard deviations, and the interquartile range (IQR). The Shapiro–Wilk test was used to assess the normality of each parameter, with a p-value ≥ 0.05 indicating normal distribution. For parameters that did not follow a normal distribution, the Mann–Whitney U-test was used to compare differences between sex and age groups. In addition, multiple linear regression analysis was performed to evaluate the effects of demographic characteristics, including sex and age, on femoral head coverage parameters (LCEA, ACEA, PCEA, AASA, and PASA). Each parameter was analyzed as a separate dependent variable, with sex and age included as independent variables. After applying the Benjamini-Hochberg procedure, statistical significance was set at p < 0.05.

## Results

### Patients

A total of 270 pelvic samples, including 158 from men and 112 from women from a single ethnic group, were included in this study. All included participants had undergone pelvic 3D CT scans, which revealed no pelvic or joint abnormalities. The age of the participants ranged from 27 to 69 years, with an average of 56.1 years and a standard deviation of 9.8. The age characteristics of the participants are summarized in Table 1.

**Table 1:** Age characteristics of the collected three-dimensional computed tomography samples.

Sex	Age*						Mean*	SD
	20–29	30–39	40–49	50–59	60–69	Total		
Male	1	13	28	52	64	158	55.3	9.8
Female	0	8	16	31	57	112	57.1	9.9
Total	1	21	44	83	121	270	56.1	9.8

SD: Standard deviation.

* In years.

### Femoral head coverage measurement

The statistical characteristics of the femoral head coverage angles measured from all 3D CT images are shown in Table 2. The mean of LCEA, ACEA, PCEA, AASA, and PASA were 33.6°, 84.4°, 96.2°, 64.1°, and 82.0°, respectively, with an interquartile range of 28.9°–38.1°, 77.5°–93.5°, 93.0°–100.3°, 56.8°–69.3°, and 81.1°–87.8°, respectively. Fig 5 shows a visualization of the mean values of the angles listed in Table 2.

**Table 2:** Femoral head coverage based on three-dimensional computed tomography images of all samples.

	LCEA	ACEA	PCEA	AASA	PASA
**Mean** ^**a**^	33.6	84.4	96.2	64.1	84.0
**Range** ^**a**^	10.7–75.2	52.6–104.6	78.0–116.9	42.5–100.4	54.6–106.7
**SD** ^**a**^	7.9	11.1	6.5	10.2	5.4
**IQR** ^**a**^	28.9–38.1	77.5–93.5	93.0–100.3	56.8–69.3	81.1–87.8

Abbreviations: LCEA, lateral center-edge angle; ACEA, anterior center-edge angle; PCEA, posterior center-edge angle; AASA, anterior acetabular sector angle; PASA, posterior acetabular sector angle; SD, standard deviation; IQR, interquartile range.

^a^The values are given in degrees.

**Fig 5:**
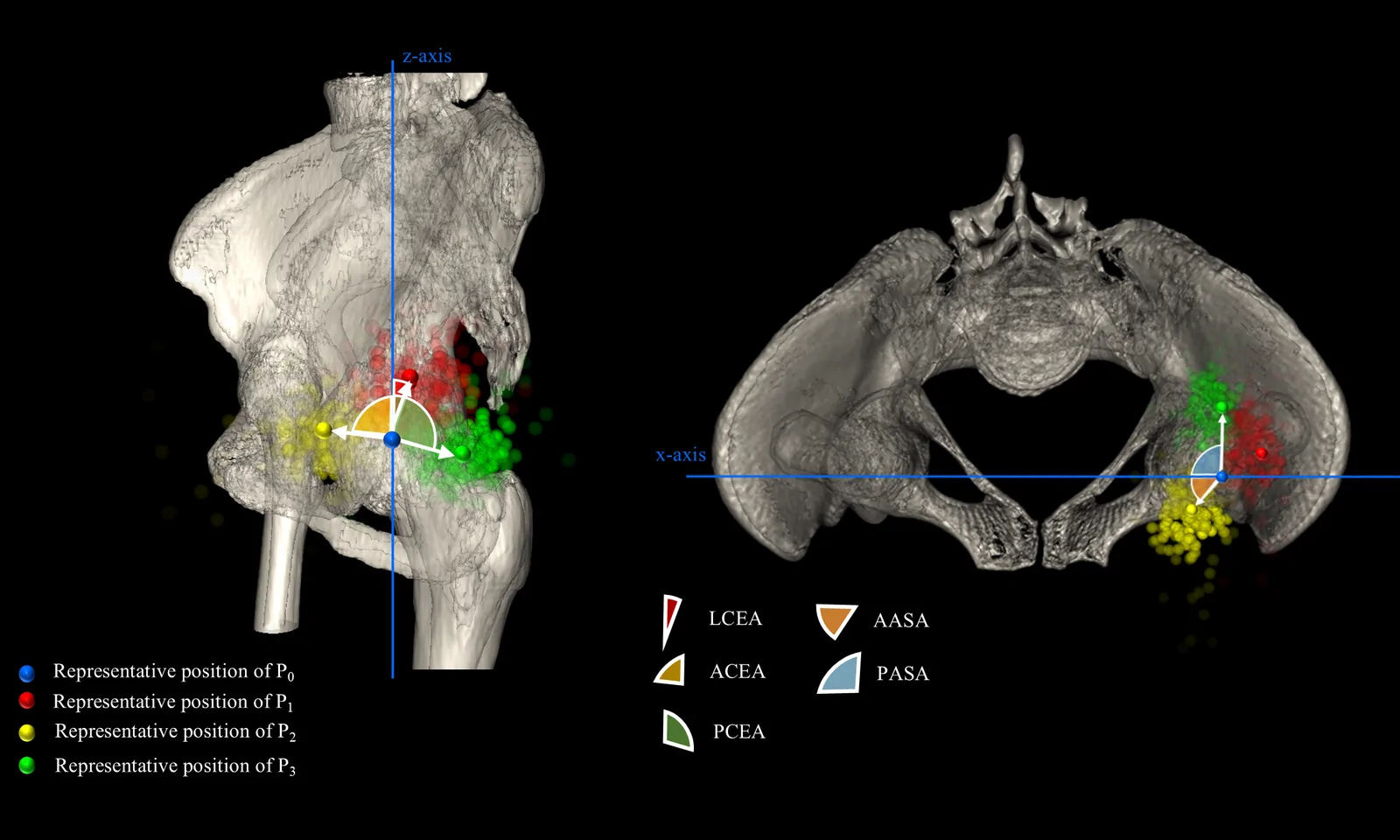
Visualization of P_0_, P_1_, P_2_, and P_3_, and the five parameters on 3D-rendered images of the pelvic bone with the bilateral femur. To enhance the visibility of the representative landmarks, the opacities of 3D-rendered images of bony structures were slightly reduced. P_0_ is indicated as a single blue point, representing the center of the spherical femoral head of the samples. The positions of P_1_, P_2_, and P_3_ of the samples are marked with low opacity. The representative positions of P_1_, P_2_, and P_3_ are indicated with full opacity. The vectors connecting the representative P_0_ and P_1_, P_2_, or P_3_ are indicated by white arrows. The z-axis- and x-axes are shown, and the angles between the axis and vector are marked: lateral center-edge angle (LCEA), anterior center-edge angle (ACEA), and posterior center-edge angle (PCEA) on the left, and anterior acetabular sector angle (AASA) and posterior acetabular sector angle (PASA) on the right.

### Sex- and age-specific differences in 3D femoral head coverage

Table 3 shows the mean and statistical characteristics of the femoral head coverage angles by sex. The mean values for LCEA, ACEA, PCEA, AASA, and PASA in men and women were 34.8° and 32.0°, 83.6° and 85.5°, 95.8° and 96.9°, 65.5° and 62.1°, and 84.1° and 83.9°, respectively. The results of the Mann-Whitney U-test revealed significant differences in LCEA and AASA between men and women, with women having lower mean values for both angles. According to the results of the multiple linear regression analysis, both sex (p = 0.001) and age (p < 0.001) were statistically significant for LCEA, sex was statistically significant for AASA (p = 0.011), and age was statistically significant for PASA (p = 0.008) (S1 Table).

**Table 3:** Femoral head coverage based on three-dimensional computed tomography images according to sex.

Sex		LCEA	ACEA	PCEA	AASA	PASA
**Male**	**Mean** ^**a**^	34.8	83.6	95.8	65.5	84.1
	**Range** ^**a**^	20.4–75.2	57.0–104.6	78.3–113.8	42.5–100.4	67.2–106.7
	**SD** ^**a**^	8.3	10.9	6.3	10.3	5.3
	**IQR** ^**a**^	29.5–39.3	77.1–92.2	92.4–99.9	57.9–71.0	81.1–88.2
**Female**	**Mean** ^**a**^	32.0	85.5	96.9	62.1	83.9
	**Range** ^**a**^	10.7–53.4	52.6–103.0	78.0–116.9	43.9–95.8	54.6–97.2
	**SD** ^**a**^	6.9	11.3	6.8	9.7	5.6
	**IQR** ^**a**^	28.3–36.1	79.4–95.4	93.5–100.7	55.3–67.2	81.1–87.5
***p*** ^**b**^		0.046*	0.233	0.248	0.028*	0.888

Abbreviations: LCEA, lateral center-edge angle; ACEA, anterior center-edge angle; PCEA, posterior center-edge angle; AASA, anterior acetabular sector angle; PASA, posterior acetabular sector angle; SD, standard deviation; IQR, interquartile range.

a The values are given in degrees.

b Statistical test was conducted using the Mann–Whitney U test and the Benjamini-Hochberg procedure was applied on raw p-values.

* Indicate statistically significant values.

Table 4 summarizes the results of the statistical analysis of the angles by age group (age <50 and >50 years). The Mann–Whitney U-test was used to analyze differences in mean values across age groups. Statistically significant differences in LCEA between the age groups were observed in men, whereas women exhibited significant differences in LCEA, ACEA, and AASA between the age groups. Although LCEA, ACEA, and PCEA increased with age, AASA and PASA showed a decreasing trend. Fig 6 shows a graphical illustration of the statistically significant differences in angles by sex and age. A regression analysis graph showing the difference in LCEA by sex and normal LCEA values across age in both sexes is shown in Fig 7.

**Table 4:** Femoral head coverage based on three-dimensional computed tomography images in males and females according to age.

Sex	Age		LCEA	ACEA	PCEA	AASA	PASA
**Male**	**< 50 years**	**Mean** ^**a**^	32.1	82.9	95.6	66.0	84.7
		**Range** ^**a**^	20.4–58.2	57.0–104.6	79.4–107.4	49.5–100.4	68.3–91.2
		**SD** ^**a**^	8.1	13.4	7.0	11.3	5.1
	**≥ 50 years**	**Mean** ^**a**^	35.8	83.8	95.9	65.3	83.9
		**Range** ^**a**^	21.6–75.2	60.5–102.3	78.3–113.8	42.5–91.3	67.2–106.7
		**SD** ^**a**^	8.2	9.9	6.1	10.0	5.4
	***p*** ^**b**^		0.028*	0.942	0.942	0.942	0.352
**Female**	**< 50 years**	**Mean** ^**a**^	28.8	80.1	96.1	66.5	84.8
		**Range** ^**a**^	16.5–38.3	57.9–103.0	78.5–116.9	48.9–95.8	78.3–95.1
		**SD** ^**a**^	5.4	12.1	8.8	10.4	3.9
	**≥ 50 years**	**Mean** ^**a**^	32.9	86.9	97.1	60.9	83.6
		**Range** ^**a**^	10.7–53.4	52.6–101.2	78.0–111.4	43.9–92.6	54.6–97.2
		**SD** ^**a**^	7.1	10.6	6.2	9.2	6.0
	***p*** ^**b**^		0.028*	0.037*	0.557	0.037*	0.942

Abbreviations: LCEA, lateral center-edge angle; ACEA, anterior center-edge angle; PCEA, posterior center-edge angle; AASA, anterior acetabular sector angle; PASA, posterior acetabular sector angle; SD, standard deviation; IQR, interquartile range.

a The values are given in degrees.

b Statistical test was conducted using the Mann–Whitney U test and the Benjamini-Hochberg procedure was applied on raw p-values.

* Indicate statistically significant values.

**Fig 6:**
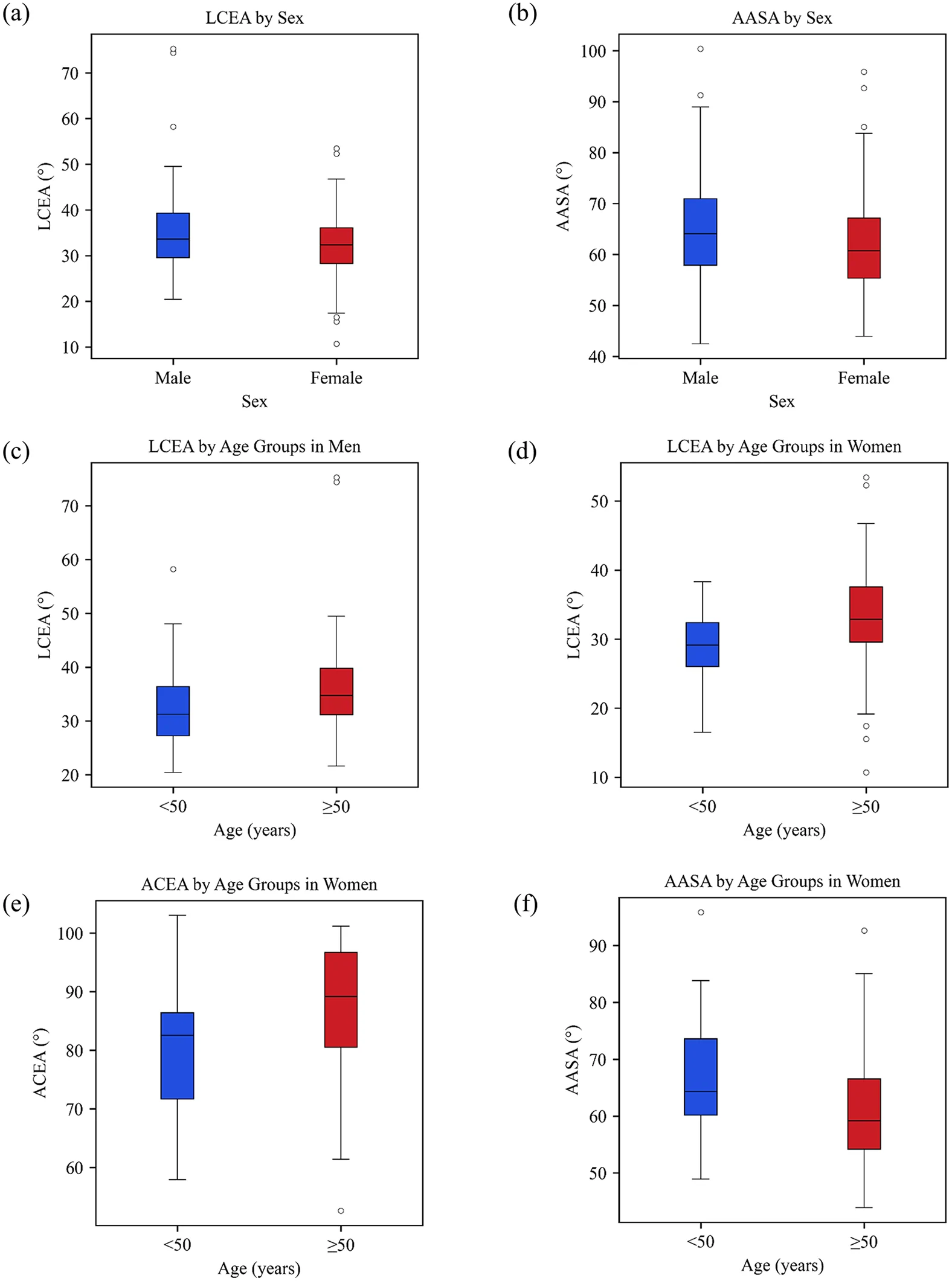
Boxplots visualizing the statistically significant differences among sexes and age groups. The angles are in degrees and the ages are in years. (a) Differences in LCEA according to sex, (b) Differences in AASA according to sex, (c) Differences in LCEA among different age groups in men, (d) Differences in LCEA among different age groups in women, (e) ACEA differences among age groups in women, (f) AASA differences among age groups in women.

**Fig 7:**
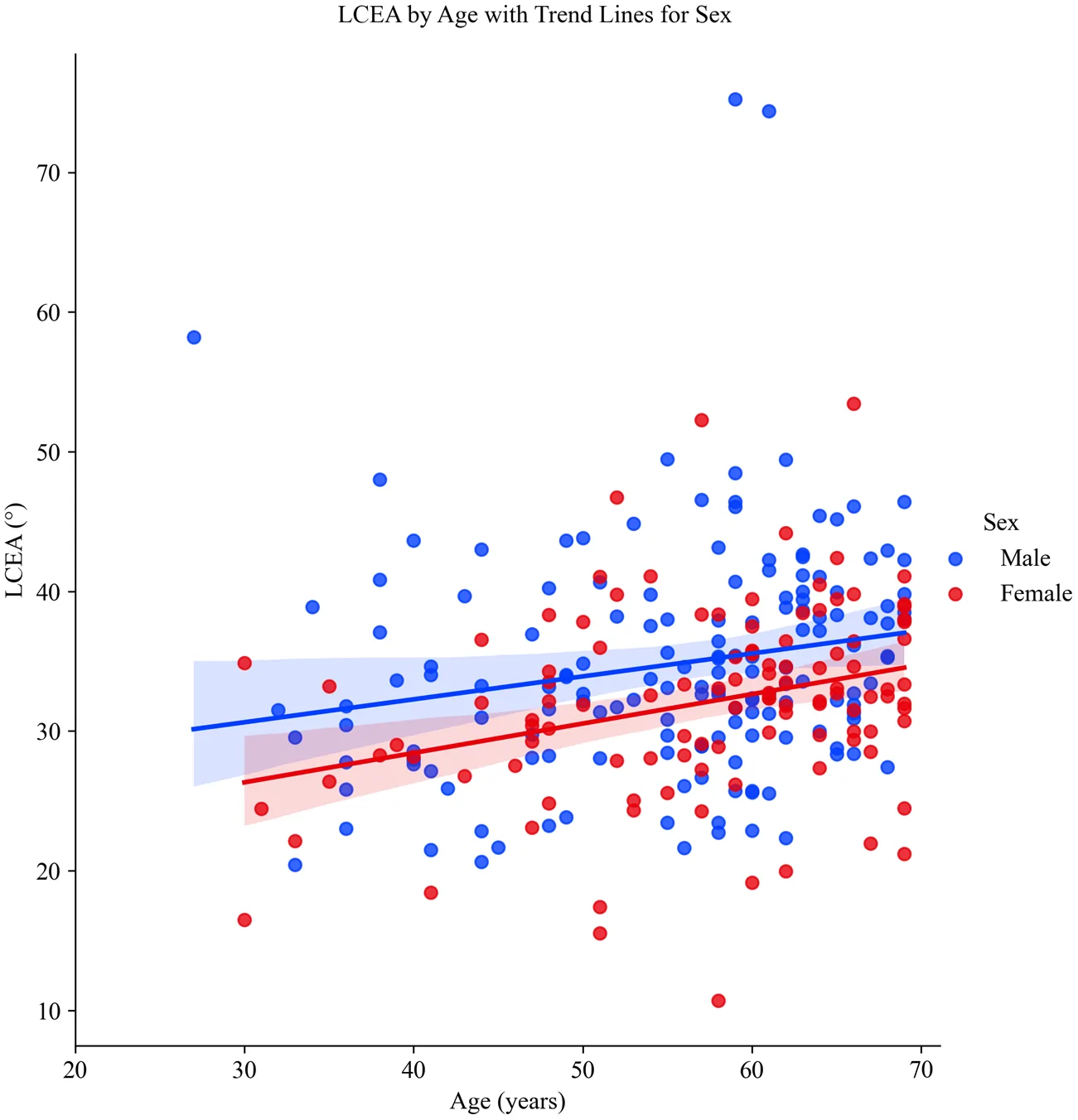
Scatter plot and trend line showing the normal values of LCEA depending on age in men and women. The angles are in degrees and the ages are in years. All the values are marked with a separate trend line for men and women. Lightly shaded areas near the trend line represent the 95% confidence interval, indicating the value for each sex depending on age.

## Discussion

This study aimed to establish standard reference values for femoral head coverage indicators measured in a three-dimensional space in a Korean population. Among the indicators, statistically significant differences between the sexes were observed for LCEA and AASA. For LCEA, the range of standard values, excluding outliers, for men and women were 29.5–39.3° and 28.3–36.1°, respectively. Similarly, the standard range for AASA was 57.9–71.0° in men and 55.3–67.2° for women. For ACEA, PCEA, and PASA, the standard values across both sexes were 77.5–93.5°, 93.0–100.3°, and 81.1–87.8°, respectively.

Given that femoral head coverage is a crucial clinical parameter for diagnosing and treating various hip joint diseases, it is essential to have detailed knowledge of its normal values. Traditionally, femoral head coverage has been measured using 2D imaging methods in clinical practice, such as analyzing specific slices from radiographs or 2D CT images to measure the coverage within a single plane. However, these measurements, particularly LCEA and AASA, are prone to potential errors when obtained using traditional 2D methods. By measuring these angles in 3D space, this study provides reference values derived in three-dimensional space. Additionally, since this study defined femoral head coverage relying on bony structures, the results are unaffected by cartilage and other soft tissues, therefore having robustness. These refined measurements are particularly useful for practical applications such as total hip replacement (THR). For instance, femoral head coverage is used as a criterion to determine whether to use a shelf autograft for cup fixation during THR and as an indicator to assess whether the cup is properly positioned postoperatively [14,25]. Comparing the values established in this study with patient-specific values can facilitate prompt surgical planning and verify outcomes both before and after surgery by providing surgeons with specific assistance for implant positioning thereby supporting surgical planning and implant positioning.

Since its first proposal by Wiberg, LCEA has been widely recognized as a traditional measure of femoral head coverage and an important diagnostic marker for structural hip diseases. Although LCEA is considered the most reliable angle measured using 2D images [26,27], its susceptibility to errors makes 3D measurements potentially more advantageous in terms of diagnosis. Additionally, it is feasible to compare our findings with those of previous studies.

Miyakasa et al. reported differences in femoral head coverage measured using 2D images between the sexes and different age groups. The average LCEA, ACEA, PCEA, AASA, and PASA values in men and women were 32.5° and 31.6°, 58.2° and 56.0°, 97.1° and 102.9°, 61.2° and 57.1°, and 94.5° and 96.8°, respectively [5]. In this study, the corresponding average values of these angles in men and women were 34.8° and 32.0°, 83.6° and 85.5°, 95.8° and 96.9°, 65.5° and 62.1°, and 84.1° and 83.9°, respectively. Differences were observed between the 2D and 3D measurements, particularly for ACEA and PASA. Miyakasa et al. measured the angles within the same slice of a CT image; however, this study used mathematical analysis to measure the angles between points that were not located on the same slice. Considering that the LCEA, AASA, and PCEA were broadly comparable between the 2D and 3D methods, it can be assumed that the lateral, anterior, and posterior margins of the acetabulum were placed in similar positions in both studies. However, the 3D measurement method may have affected the results. Furthermore, the standard deviations of the five angles in the previous study ranged from 4.2° to 16.2°, whereas they ranged from 3.9° to 13.4° in this study. This suggests that the values measured in this study are fairly consistent,.

The similarities and differences between the results of this study and those of previous studies conducted in other ethnic groups should be highlighted. Hawellek et al. reported standard LCEA values of 40.3° in men and 41.8° in women in a German population [6]. Shi et al. reported standard LCEA values of 28.0° for male individuals and 28.4° for female individuals in a Chinese population [7]. Similarly, Umer et al. reported LCEA values of 30.6° for men and 33.5° for women in their study on the Singaporean population [8]. The standard values presented in this study were 34.8° for men and 32.0° for women, which are comparable to the values from other Asian populations rather than those from the German population. Given the ethnic proximity of the Chinese and Singaporean populations to the Korean population examined in this study, similar LCEA values observed reinforce the idea that there is a difference in femoral head coverage between the Western and Asian populations.

However, the range of standard values for LCEA measured in this study differed from the traditionally recognized standard for LCEA. In 2D measurements, an LCEA of less than 20° is considered indicative of hip dysplasia [20,28]; however, our 3D analysis identified minimum LCEA values as low as 10.7°. Smaller values were measured in patients with clinically normal pelvis and joints, and the standard deviation of the LCEA within the group was up to 10.0, which was not significantly different from the values reported in previous studies. Therefore, both the minimum and maximum values should be considered statistically significant. However, it cannot be definitively concluded that the minimum value necessarily indicates dysplasia. This difference in 2D and 3D measurements may be due to differences in geometric projection and anatomical reference points. Therefore, direct comparison between 2D and 3D angle measurements should be interpreted with caution, and further studies are needed to establish diagnostic thresholds specific to 3D imaging.

Among the five angles measured in this study, the LCEA showed a particularly significant difference among the age groups. Statistically significant differences were observed between individuals aged <50 and ≥ 50 years in both sexes. The average LCEA values were higher in men than in women, and the average value increased with increasing age in both sexes. LCEA increases during growth [29]; however, changes in LCEA among different age groups in adults remain unclear, albeit several studies have reported an age-related increase [6,30]. Despite limited research on the underlying cause, it can be hypothesized that as the joint ages, the stress exerted on the acetabulum by the femoral head accumulates, leading to gradual expansion of the acetabulum.

In addition to LCEA, ACEA and AASA showed significant differences in average values across different age groups in women. The trend for ACEA was generally consistent with that of LCEA, with smaller values observed in women aged <50 years than in those aged >50 years. However, the trend for AASA showed an inverse pattern, with higher values observed in younger women aged <50 years. This difference in trends could be attributed to the measurement of AASA along the horizontal axis. Xiong et al. demonstrated that during walking, the pressure within contact of the bones is mostly concentrated at the superior part of the femoral head and dome of the acetabulum [31]. This suggests that, while standing and walking, the acetabulum receives stronger forces in the vertical direction, implying that the anterior and posterior margins may not be subjected to strong forces compared to that of the lateral margin. The forces concentrated on the anterior and posterior margins may not be sufficient to compensate for the natural processes of bone wear and weakening of the joints, leading to smaller sector angles.

Although this study provided valuable reference values, there were certain limitations. First, the number of observations, particularly the number of 3D CT images of younger adults, was relatively small. In most clinical cases, 3D CT scans of the pelvis are performed when abnormalities or fractures are suspected. Consequently, a limited number of individuals who underwent pelvic 3D scans over a 10-year period met the inclusion criteria for this study. Additionally, as older individuals are more likely to require 3D CT imaging due to bone weakening or joint degeneration, more than 44% of the observed normal cases were from individuals aged ≥ 60 years, leading to an underrepresentation of younger individuals. This bias may restrict generalizability to the broader population. Since data were collected and analyzed retrospectively, the sample in this study reflects the clinical population undergoing 3D CT evaluation that does not involve anatomical abnormalities. Second, owing to the limitations in CT imaging, accurately determining the vertical and horizontal axes of the pelvis remained challenging. Ideally, the vertical axis should be defined as the line connecting the center of the femoral head and femoral posterior condyle, whereas the horizontal axis should connect the centers of both femoral heads [32]. However, it was difficult to designate the axes in this way, given the narrow scope and the complexity of 3D CT images. Third, landmark identification was performed by two board-certified orthopedic surgeons through simultaneous consensus rather than independent measurement. Consequently, inter-observer reliability (e.g., intraclass correlation coefficient) could not be quantified, and future studies employing independent measurements are warranted to formally assess the reproducibility of the 3D technique. For future research, study design with larger samples of younger patients will expand applicability. Automating the measuring process of 3D markers would also be clinically significant. Comparison between the traditional method of measuring femoral health coverage on 2D images and the 3D method on their ability to diagnose pelvic abnormalities could also be explored. Future studies may also incorporate artificial intelligence such as machine learning to predict patient outcomes or diagnoses using 3D markers.

The 3D femoral head coverage angle standards for Koreans presented in this study were designed for orthopedic diagnosis and treatment. Given the extensive application of 3D medical imaging, the standard values provided in this study may be beneficial for diagnosing hip joint conditions and designing medical devices such as prosthetics for surgical interventions.

## Conclusion

To address the limitations of femoral head coverage measurements in 3D, there is a need for reference values derived from 3D imaging, particularly given the limited research on the morphology of the Korean acetabulum. In this study, we used 3D pelvic CT images from healthy individuals to define key anatomical landmarks, the center, lateral, anterior, and posterior margins of the acetabulum, and measured the femoral head coverage angles in 3D space. The normal values for 3D LCEA in Koreans are 34.8° (SD = 8.3) for men and 32.0° (SD = 6.9) for women. Furthermore, our findings revealed that the 3D angles differed significantly according to sex and age. The center-edge angles such as the LCEA, ACEA, and PCEA tended to increase with age, whereas the sector angles such as the AASA and PASA showed the opposite trend. The standard values and statistical analyses based on sex and age presented in this study will provide practical guidance for the surgical treatment of the acetabulum.

## Supporting information

S1 Table Multiple linear regression of femoral head coverage angles on sex and age.
(DOCX)

## References

[1] Henebry A, Gaskill T. The effect of pelvic tilt on radiographic markers of acetabular coverage. Am J Sports Med. 2013;41(11):2599–603. doi: 10.1177/0363546513500632 23982398

[2] Lommerse MI, Willems HC, van Dieren S, Bloemers FW, Schuijt HJ, van Embden D. Increasing incidences of acetabular, pelvic, and proximal femur fractures in The Netherlands. Injury. 2025;56(6):112322. doi: 10.1016/j.injury.2025.112322 40198969

[3] Park K-T, Park EK, Lee D-H, An JH, Won J, Kim SH, et al. The Current Incidence and Future Projection of Acetabular Fractures in Korea. J Korean Med Sci. 2024;39(27):e204. doi: 10.3346/jkms.2024.39.e204 39015002 PMC11249580

[4] Bakhshayesh P, Weidenhielm L, Enocson A. Factors affecting mortality and reoperations in high-energy pelvic fractures. Eur J Orthop Surg Traumatol. 2018;28(7):1273–82. doi: 10.1007/s00590-018-2203-1 29675632 PMC6132923

[5] Miyasaka D, Sakai Y, Ibuchi S, Suzuki H, Imai N, Endo N. Sex- and age-specific differences in femoral head coverage and acetabular morphology among healthy subjects-derivation of normal ranges and thresholds for abnormality. Skeletal Radiol. 2017;46(4):523–31. doi: 10.1007/s00256-017-2583-z 28150099

[6] Hawellek T, Meier M-P, Seitz M-T, Uhlig J, Hosseini ASA, Beil FT, et al. Morphological Parameters of the Hip Joint and Its Relation to Gender, Joint Side and Age-A CT-Based Study. Diagnostics (Basel). 2022;12(8):1774. doi: 10.3390/diagnostics12081774 35892485 PMC9332566

[7] Shi YY, Liu TJ, Zhao Q, Zhang LJ, Ji SJ, Wang EB. The normal centre-edge angle of Wiberg in the Chinese population. J Bone Jt Surg Br Vol. 2010;92-B(8):1144–7. doi: 10.1302/0301-620x.92b8.23993 20675762

[8] Umer M, Thambyah A, Tan WT, Das De S. Acetabular morphometry for determining hip dysplasia in the Singaporean population. J Orthop Surg (Hong Kong). 2006;14(1):27–31. doi: 10.1177/230949900601400107 16598083

[9] Tuğrul Aİ, Yılmaz G, Aydın BK, Akel İ, Durgut F, Şenaran H. Center-edge angle values in healthy children between 5 and 14 years old in Turkey. Acta Orthop Traumatol Turc. 2020;54(1):15–9. doi: 10.5152/j.aott.2020.01.451 32175892 PMC7243687

[10] Edwards K, Leyland KM, Sanchez-Santos MT, Arden CP, Spector TD, Nelson AE, et al. Differences between race and sex in measures of hip morphology: a population-based comparative study. Osteoarthritis Cartilage. 2020;28(2):189–200. doi: 10.1016/j.joca.2019.10.014 31843571

[11] Ogata S, Moriya H, Tsuchiya K, Akita T, Kamegaya M, Someya M. Acetabular cover in congenital dislocation of the hip. J Bone Joint Surg Br. 1990;72(2):190–6. doi: 10.1302/0301-620X.72B2.2312554 2312554

[12] Pullen WM, Henebry A, Gaskill T. Variability of acetabular coverage between supine and weightbearing pelvic radiographs. Am J Sports Med. 2014;42(11):2643–8. doi: 10.1177/0363546514548854 25214530

[13] Uemura K, Atkins PR, Peters CL, Anderson AE. The effect of pelvic tilt on three-dimensional coverage of the femoral head: A computational simulation study using patient-specific anatomy. Anat Rec (Hoboken). 2021;304(2):258–65. doi: 10.1002/ar.24320 31755243 PMC7311050

[14] Mancino F, Fontalis A, Magan A, Plastow R, Haddad FS. The Value of Computed Tomography Scan in Three-dimensional Planning and Intraoperative Navigation in Primary Total Hip Arthroplasty. Hip Pelvis. 2024;36(1):26–36. doi: 10.5371/hp.2024.36.1.26 38420736 PMC10929541

[15] Hassani H, Cherix S, Ek ET, Rüdiger HA. Comparisons of preoperative three-dimensional planning and surgical reconstruction in primary cementless total hip arthroplasty. J Arthroplasty. 2014;29(6):1273–7. doi: 10.1016/j.arth.2013.12.033 24502952

[16] Sariali E, Mauprivez R, Khiami F, Pascal-Mousselard H, Catonné Y. Accuracy of the preoperative planning for cementless total hip arthroplasty. A randomised comparison between three-dimensional computerised planning and conventional templating. Orthop Traumatol Surg Res. 2012;98(2):151–8. doi: 10.1016/j.otsr.2011.09.023 22381566

[17] Martin CM, Turgeon JG, Goela A, Rice CL, Wilson TD. A three-dimensional measurement approach for the morphology of the femoral head. J Anat. 2014;225(3):358–66. doi: 10.1111/joa.12207 24952992 PMC4166976

[18] Upasani VV, Bomar JD, Bandaralage H, Doan JD, Farnsworth CL. Assessment of three-dimensional acetabular coverage angles. J Hip Preserv Surg. 2020;7(2):305–12. doi: 10.1093/jhps/hnaa026 33163216 PMC7605764

[19] Hanson JA, Kapron AL, Swenson KM, Maak TG, Peters CL, Aoki SK. Discrepancies in measuring acetabular coverage: revisiting the anterior and lateral center edge angles. J Hip Preserv Surg. 2015;2(3):280–6. doi: 10.1093/jhps/hnv041 27011850 PMC4765297

[20] Wiberg G. Studies on Dysplastic Acetabula and Congenital Subluxation of the Hip Joint with Special Reference to the Complication of Osteo-Arthritis. J Am Med Assoc. 1940;115(1):81. doi: 10.1001/jama.1940.02810270083038

[21] Ibuchi S, Imai N, Horigome Y, Suzuki H, Kawashima H. The Three-Dimensional Criteria of Developmental Dysplasia of the Hip Using the Functional Pelvic Plane Is More Useful Than That Using the Anterior Pelvic Plane. J Clin Med. 2024;13(9):2536. doi: 10.3390/jcm13092536 38731066 PMC11084296

[22] Warming L, Hassager C, Christiansen C. Changes in bone mineral density with age in men and women: a longitudinal study. Osteoporos Int. 2002;13(2):105–12. doi: 10.1007/s001980200001 11905520

[23] Kim Y, Lee K-W, Lee S, Woo EJ, Hu K-S. Age-related morphological changes of the pubic symphyseal surface: using three-dimensional statistical shape modeling. Sci Rep. 2025;15(1):494. doi: 10.1038/s41598-024-84168-8 39747495 PMC11696140

[24] Imai N, Suzuki H, Nozaki A, Miyasaka D, Tsuchiya K, Ito T, et al. Evaluation of anatomical pelvic parameters between normal, healthy men and women using three-dimensional computed tomography: a cross-sectional study of sex-specific and age-specific differences. J Orthop Surg Res. 2019;14(1):126. doi: 10.1186/s13018-019-1165-2 31072379 PMC6506943

[25] Taylor AJ, Runner RP, Kay RD, Najibi S. Femoral head autograft can reliably reconstruct dysplastic acetabula through the direct anterior approach for total hip arthroplasty. Arthroplast Today. 2022;14:154–62. doi: 10.1016/j.artd.2022.02.005 35313717 PMC8933727

[26] Uemura K, Hiraiwa T, Okamoto M, Tokunaga K, Anderson AE. The anterior center edge angle has limited ability to predict three-dimensional coverage of the femoral head in patients with developmental dysplasia of the hip undergoing curved periacetabular osteotomy. Arch Orthop Trauma Surg. 2023;143(3):1323–30. doi: 10.1007/s00402-021-04258-4 34977962 PMC9250550

[27] Zhang D, Pan X, Zhang H, Luo D, Cheng H, Xiao K. The lateral center-edge angle as radiographic selection criteria for periacetabular osteotomy for developmental dysplasia of the hip in patients aged above 13 years. BMC Musculoskelet Disord. 2020;21(1):493. doi: 10.1186/s12891-020-03515-8 32711501 PMC7382803

[28] Werner CML, Ramseier LE, Ruckstuhl T, Stromberg J, Copeland CE, Turen CH, et al. Normal values of Wiberg’s lateral center-edge angle and Lequesne’s acetabular index--a coxometric update. Skeletal Radiol. 2012;41(10):1273–8. doi: 10.1007/s00256-012-1420-7 22584462

[29] Fredensborg N. The CE angle of normal hips. Acta Orthop Scand. 1976;47(4):403–5. doi: 10.3109/17453677608988709 961394

[30] Hofmann U-K, Ipach I, Rondak I-C, Syha R, Götze M, Mittag F. Influence of Age on Parameters for Femoroacetabular Impingement and Hip Dysplasia in X-Rays. Acta Ortop Bras. 2017;25(5):197–201. doi: 10.1590/1413-785220172505173951 29081704 PMC5608738

[31] Xiong B, Yang P, Lin T, Xu J, Xie Y, Guo Y, et al. Changes in hip joint contact stress during a gait cycle based on the individualized modeling method of “gait-musculoskeletal system-finite element”. J Orthop Surg Res. 2022;17(1):267. doi: 10.1186/s13018-022-03094-5 35568957 PMC9107226

[32] DeFroda SF, Alter TD, Lambers F, Malloy P, Clapp IM, Chahla J, et al. Quantification of Acetabular Coverage on 3-Dimensional Reconstructed Computed Tomography Scan Bone Models in Patients With Femoroacetabular Impingement Syndrome: A Descriptive Study. Orthop J Sports Med. 2021;9(11):23259671211049457. doi: 10.1177/23259671211049457 34820460 PMC8607491

