# Supplementary material for: Normal values of femoral head coverage angles on three-dimensional computed tomography images
Source: PLoS One. 2026 Oct 7;21(10):e0347008. doi: 10.1371/journal.pone.0347008 (PMC13645054; doi:10.1371/journal.pone.0347008)
Supplement: Supplementary file 1 [file pone.0347008.s001.docx]

# **Supplementary**

**S1 Table.** Multiple linear regression of femoral head coverage angles on sex and age

| **Parameter** | **Predictor** | ***β*** | **95% CI** | ***p*** |
| --- | --- | --- | --- | --- |
| LCEA  *(R^2^ = 0.082)* | Sex (M) | 3.08 | 1.23 to 4.93 | 0.001 |
|  | Age | 0.18 | 0.09 to 0.28 | <0.001 |
| ACEA  *(R^2^ = 0.018)* | Sex (M) | −1.70 | −4.39 to 0.99 | 0.214 |
|  | Age | 0.12 | −0.02 to 0.25 | 0.087 |
| PCEA  *(R^2^ = 0.013)* | Sex (M) | −0.98 | −2.57 to 0.61 | 0.225 |
|  | Age | 0.05 | −0.03 to 0.14 | 0.177 |
| AASA  *(R^2^ = 0.040)* | Sex (M) | 3.17 | 0.73 to 5.61 | 0.011 |
|  | Age | −0.12 | −0.24 to 0.00 | 0.059 |
| PASA  *(R^2^ = 0.026)* | Sex (M) | 0.08 | −1.23 to 1.39 | 0.905 |
|  | Age | −0.09 | −0.16 to −0.02 | 0.008 |

*β, unstandardized regression coefficient; CI, confidence interval. All models were fitted with sex and age as predictors (N = 270). Sex was coded with female as the reference category; the coefficient for Sex (M) represents the difference of males relative to females.*
